# Supplementary material for: Association between Dietary Inflammatory Index and Type 2 diabetes mellitus in Xinjiang Uyghur autonomous region, China
Source: PeerJ. 2021 Jul 16;9:e11159. doi: 10.7717/peerj.11159 (PMC8288110; doi:10.7717/peerj.11159)
Supplement: File S3 [file peerj-09-11159-s003.pdf]

National key research and development plan "Precision medicine research" key special northwest region natural population queue research

Study on cohort construction and health follow-up of natural multiethnic population in Xinjiang

Subject Questionnaire

Physical examination number：

Baseline Questionnaire

Name：		Sex：①Male ②Female
Age：	— —years	Date: __Year__month__day

The survey process

NO.	content	Sign√ when finished
1	Registration form, informed consent	☐
2	Collection of blood specimens	☐
3	Measure Blood pressure	☐
4	Measure height, waist circumference	☐
5	Measure weight, body fat	☐
6	Consultation investigation	☐
7	Table check	☐

Medical examination items

1.1 Height（**cm**） — — —.—

1.2 Waist（**cm**） — — —.—

1.3 Heart rate（Times/min） — — —

1.4 Blood pressure（**mmHg**）

	Systolic pressure	Diastolic pressure
First time	— — —.—	— — —.—
Second time	— — —.—	— — —.—

1.5 Weight（**kg**） — — —.—

1.6 Body fat composition

Body fat rate（**%**） — — —.—

Body fat（**kg**） — — —.—

Muscle mass（**kg**） — — —.—

1.General information（Please tick √ on the appropriate box ○，Please fill in the information in the□as require）

1.1 Physical examination number：（See cover）

1.2 The name of your organization：______________________________

1.3 Sex：（See cover） ○ Male ○ Female

1.4 Date of birth：□□□□ year□□month□□day

1.5 ID number：□□□□□□□□□□□□□□□□□□

1.5.1 Medicare number：□□□□□□□□□□□□□□□□□□

1.6 Home Address：Xinjiang Uygur Autonomous Region（City/Autonomous Prefecture) ________________________ Streets/towns ______________________Neighborhood committee/village

1.7 Cell phone number：□□□□□□□□□□□

1.7.1 Important contact phone number：□□□□□□□□□□□

1.8 Your national

○ Han ○ Hui

○ Uyghur ○ Kazak

○ Other（**1.8a**）________

1.9 What is the highest education you have received?

○Had no formal schooling ○Junior college

○Primary school ○College

○Junior high school ○Graduate or above

○High schools (including technical/technical schools)

1.10 What was your family's total income (including all sources) last year？

○ <2,500 yuan ○35,000-49,900 yuan

○2,500-4,999 yuan ○50,000-74,900 yuan

○5,000-9,999 yuan ○75,000-999,000 yuan

○10,000-19,900 yuan ○100,000-199,000 yuan

○20,000-34,900 yuan ○≥200,000 yuan

2. Tea and coffee consumption（Please tick √ on the appropriate boxO，Please fill in the information in the ☐ as require）

2.1 How often have you had tea in the past year?

O never or almost never drink

O only on special occasions (such as holidays) drink occasionally

O drink only on special season of a year（Such as busy farming or summer）

O regardless of season, every month to drink, but the frequency less than once a week

O regardless of season, basically every week

2.2 How often have you had coffee in the past year?

O Never or almost never drink coffee

O only on special occasions（Such as holidays）drink occasionally

O drink every month, but the frequency less than once a week

O basically, drink every week

2.3 When did you start the habit of drinking coffee every week? ☐ ☐ year

3. Drinking（Please tick √ on the appropriate box O）

3.1 How often have you had a drink in the past year?

O never or almost never drink

O only on special occasions (such as holidays) drink occasionally

O drink only on special season of a year（Such as busy farming or summer）

O regardless of season, every month to drink, but the frequency less than once a week

O regardless of season of a year, basically every week

4.Smoking（Please tick √ on the appropriate boxO，Please fill in the information in the☐ as require）

4.1 How often do you smoke now?

ONever Ooccasionally Omost days Oevery day

4.2 If you used to smoke but have stopped, how long have you quit? ☐☐year☐☐month

4.3 What is the most important reason for you to quit smoking?（Please choose the main answer）

Obecause suffer from diseases

O Family against

Oworry affect the health of the future (not yet ill)

Odoctor's suggestion

Odue to the economic burden

Oother

4.4 When do you start the habit of smoking every day or almost every day? ☐☐year

4.5 Have you ever tried to quit smoking（It must last at least one week）? O Yes ONo

5. Dietary situation（Please tick √ on the appropriate box○，Please fill in the information in the____ as require）

5.1 In the past year, how often did you eat the following foods or drinks? (Please select one in each row to √， and fill in the intake, if the intake is not clear, please enter 999)

	**Everyday**	**4-6 times/week**	**1-3 times/week**	**1-3 times/month**	**No/ very little**	**amount of each serving**
**staple food**	○	○	○	○	○	___liang/time
rice	○	○	○	○	○	___liang/time
millet	○	○	○	○	○	___liang/time
noodles	○	○	○	○	○	___liang/time
steamed bun	○	○	○	○	○	___liang/time
deep-fried dough cake	○	○	○	○	○	___liang/time
deep-fried dough stick	○	○	○	○	○	___liang/time
coarse cereals	○	○	○	○	○	___liang/time
**animal food**						
pork	○	○	○	○	○	___liang/time
mutton	○	○	○	○	○	___liang/time
shashlik	○	○	○	○	○	___liang/time
beef	○	○	○	○	○	___liang/time
beef jerky	○	○	○	○	○	___liang/time
horse meat	○	○	○	○	○	___liang/time
chicken	○	○	○	○	○	___liang/time
duck	○	○	○	○	○	___liang/time
pigeon meat	○	○	○	○	○	___liang/time
prawn	○	○	○	○	○	___liang/time
Fish (Labeled species)	○	○	○	○	○	___liang/time
egg	○	○	○	○	○	___liang/time

duck egg	○	○	○	○	○	___liang/time
Quail eggs (spiced)	○	○	○	○	○	___liang/time
Visceral animal	○	○	○	○	○	___liang/time
lamb liver	○	○	○	○	○	___liang/time
beef liver	○	○	○	○	○	___liang/time
chicken liver	○	○	○	○	○	___liang/time

vegetable food

fresh vegetables	○	○	○	○	○	___liang/time
carrot	○	○	○	○	○	___liang/time
white radish	○	○	○	○	○	___liang/time
radish	○	○	○	○	○	___liang/time
green turnip	○	○	○	○	○	___liang/time
long bean	○	○	○	○	○	___liang/time
eggplant	○	○	○	○	○	___liang/time
tomato	○	○	○	○	○	___liang/time
chili	○	○	○	○	○	___liang/time
pumpkin	○	○	○	○	○	___liang/time
garlic	○	○	○	○	○	___liang/time
green Chinese onion	○	○	○	○	○	___liang/time
onion	○	○	○	○	○	___liang/time
ginger	○	○	○	○	○	___liang/time
turmeric	○	○	○	○	○	___liang/time
celery cabbage	○	○	○	○	○	___liang/time
broccoli	○	○	○	○	○	___liang/time
spinach	○	○	○	○	○	___liang/time
coriander	○	○	○	○	○	___liang/time
pepper	○	○	○	○	○	___liang/time

potato	○	○	○	○	○	___liang/time
vermicelli	○	○	○	○	○	___liang/time
sweet potato	○	○	○	○	○	___liang/time
soybean	○	○	○	○	○	___liang/time
soybean milk powder	○	○	○	○	○	___liang/time
bean curd	○	○	○	○	○	___liang/time
skin of soya-bean milk	○	○	○	○	○	___liang/time
mung bean	○	○	○	○	○	___liang/time
Broad bean	○	○	○	○	○	___liang/time
chickpea	○	○	○	○	○	___liang/time
**Fresh fruits**	○	○	○	○	○	___liang/time
apple	○	○	○	○	○	___liang/time
pear	○	○	○	○	○	___liang/time
grape	○	○	○	○	○	___liang/time
orange	○	○	○	○	○	___liang/time
banana	○	○	○	○	○	___liang/time
**Dairy products**						
milk	○	○	○	○	○	___ml/time
mare's milk	○	○	○	○	○	___ml/time
ewe's milk	○	○	○	○	○	___ml/time
Yogurt (solidified/liquid)	○	○	○	○	○	___ml/time
Cream	○	○	○	○	○	___liang/time
butter	○	○	○	○	○	___liang/time
Vrum	○	○	○	○	○	___liang/time
tea with milk	○	○	○	○	○	___ml/time
buttered tea	○	○	○	○	○	___ml/time
**Other food**						
pickles	○	○	○	○	○	___liang/time

dried vegetable	○	○	○	○	○	___liang/time
nut	○	○	○	○	○	___liang/time
walnut	○	○	○	○	○	___liang/time
peanut	○	○	○	○	○	___liang/time
red dates	○	○	○	○	○	___liang/time
Matrimony vine	○	○	○	○	○	___liang/time
raisin	○	○	○	○	○	___liang/time
Other dried fruit	○	○	○	○	○	___liang/time
Candy and Chocolate	○	○	○	○	○	___liang/time
cakes and pastries	○	○	○	○	○	___liang/time

<u>Soft drinks</u>

soybean milk	○	○	○	○	○	___ml/time
Pure juice/vegetable juice （No added sugar）	○	○	○	○	○	___ml/time
sodas	○	○	○	○	○	___ml/time
Other sugary drinks	○	○	○	○	○	___ml/time
Wine (indicating category)	○	○	○	○	○	___ml/time
coffee	○	○	○	○	○	___ml/time
Red/green tea	○	○	○	○	○	___ml/time

<u>Mushroom fungus</u>

needle mushroom	○	○	○	○	○	___liang/time
Porcini mushrooms	○	○	○	○	○	___liang/time
mushroom	○	○	○	○	○	___liang/time
agaric	○	○	○	○	○	___liang/time
dried mushroom	○	○	○	○	○	___liang/time
kelp	○	○	○	○	○	___liang/time
nori	○	○	○	○	○	___liang/time

5.2 What the difference between your favorite tastes compare to those of your friends or colleagues?

○very light ○not salty not light ○ partial salty taste

5.3 In the past year, approximately how often have you been present with the following food/diet？

characteristics food	Everyday	4-6 times/week	1-3 times/week	1-3 times/month	No/ very little	amount of each serving
naan bread	○	○	○	○	○	___ml/time
Assorted Chinese Herbal Tea	○	○	○	○	○	___ml/time
herb tea	○	○	○	○	○	___ml/time
saffron crocus	○	○	○	○	○	___liang/time
**Grease**						
animal oil	○	○	○	○	○	___liang/time
colza oil	○	○	○	○	○	___liang/time
peanut oil	○	○	○	○	○	___liang/time
soya-bean oil	○	○	○	○	○	___liang/time
linseed oil	○	○	○	○	○	___liang/time
blend oil	○	○	○	○	○	___liang/time
sunflower seed oil	○	○	○	○	○	___liang/time
corn oil	○	○	○	○	○	___liang/time
olive oil	○	○	○	○	○	___liang/time
tea-seed oil	○	○	○	○	○	___liang/time

**Specific eating behavior**	Everyday	4-6 times/week	1-3 times/week	1-3 times/month	No/ very little
snack	○	○	○	○	○
Eat instant noodles/convenient rice noodles	○	○	○	○	○
night snack	○	○	○	○	○
bacon/bacon	○	○	○	○	○
ham/sausage and other processing	○	○	○	○	○
skip breakfast	○	○	○	○	○
Eat Fried food	○	○	○	○	○

Eat barbecue food	○	○	○	○	○
Eat Western fast food)	○	○	○	○	○
Eat in a restaurant or snack bar	○	○	○	○	○

5.4 In the past year, have you taken any of the following supplements for at least one month？

no	yes	
○	○	Fish oil
○	○	vitamin
○	○	Calcium/iron/zinc
○	○	Ginseng
○	○	other

5.5 How often have you eaten spicy food in the past year?

○ Never

○Occasionally, but less than once a week

} ➔ **To select these two items, go to question 5.7.**

○1 to 2 times per week

○ 3-5 times a week

○ Every day or almost every day

5.6 When do you start eating spicy food every week？ ____ year

5.7 How often have you eaten vinegar in the past year？

○Never

○ Occasionally, but less than once a week

} ➔If these two items are selected, the dietary survey is now over.

○ 1 to 2 times per week

○3-5 times a week

○Every day or almost every day

5.8 When you had the habit of eating vinegar？ ___ year

5.9 What do you think of your preference for vinegar?

○ very much ○like better ○common ○Dislike

6. Personal and family health status（Please tick √ on the appropriate box○，Please fill in the information in the____ as require）

6.1 What is your present state of health?

6.1.1 Self-Evaluation？

- well ○
- better ○
- common ○
- poor ○

6.1.2 Compare yourself to your peers？

- better ○
- same ○
- worse ○
- unaware ○

6.2 How many times have you been hospitalized due to illness in the past year?(If not, please fill in 00)

________times

6.3 Have you ever been diagnosed by a doctor in a village/district hospital or above with any of the following diseases? ○ Yes ○ No

Disease	Are you sick? Age at first diagnosis?		receive treatment？	Is still receiving medical treatment	Have you ever been in hospital? If yes, date of last hospitalization	
Diabetes	○	________	○	○	○	________
Acute myocardial infarction (a type of coronary heart disease)	○	________	○	○	○	________
Angina pectoris (a form of coronary heart disease)	○	________	○	○	○	________
Other ischemic heart attacks stroke/stroke in infants	○	________	○	○	○	________
hypertension	○	________	○	○	○	________
pulmonary heart disease	○	________	○	○	○	________
rheumatic heart disease	○	________	○	○	○	________
tuberculosis	○	________	○	○	○	________
emphysema	○	________	○	○	○	________
chronic bronchitis	○	________	○	○	○	________
chronic obstructive pulmonary disease	○	________	○	○	○	________
asthma	○	________	○	○	○	________
Chronic hepatitis/cirrhosis	○	________	○	○	○	________
peptic ulcer	○	________	○	○	○	________
Gallstones/cholecystitis	○	________	○	○	○	________
chronic nephrosis	○	________	○	○	○	________
osteoporosis	○	________	○	○	○	________
fracture	○	________	○	○	○	________
rheumatic arthritis	○	________	○	○	○	________
depressive disorder	○	________	○	○	○	________

anxiety	○	______	○	○	○	______
neurasthenia	○	______	○	○	○	______
Other mental disorders	○	______	○	○	○	______
cerebral trauma	○	______	○	○	○	______
Other	○	______	○	○	○	______
malignant tumor *	○	______	○	○	○	______

* If there is a tumor, please indicate the specific site in the box：（If there is more than one site, please select the site of the initial tumor）

○ lung ○ esophagus ○ stomach ○ liver ○ intestines ○ breast ○ prostate ○ cervix uteri ○ other

7. Physical activity（Please tick √ on the appropriate boxO，Please fill in the information in the____ as require）

7.1 In the past year, What kind of activity does your job focus on ？

O A sedentary occupation (e.g. an administrator, a secretary, etc.)

O To stand primarily (e.g. shop assistant, guard, etc.)

O General physical work

O To heavy physical labor mainly

O Retired or housework, unemployed for more than one year or physically disabled cannot work normally

7.2 How many days a week do you work on average? ____ day

7.3 How many hours do you work on average every day? ____ hour

7.4 In the past year, how did you usually get to or from work?

O walk O by motorcycle

O by bike O Private car/For hire

O Public transport (buses, subways, ferries)

O Usually works at home or near home

7.5 How much time do you spend commuting each day to and from work? ____minutes

7.6 In the past year, how often did you take part in physical exercise in your spare time?

O never or almost never attend

O 1-3 times a month

O 1-2 times a week

O 3-5 times a week

O Exercise every day or almost every day

7.7 What is your most common form of exercise？

O walking/yoga

O Running/aerobics

O Ball games (basketball, table tennis, badminton, etc.)

O Go for a brisk walk/Yanko dance/square dance

O others

7.8 What is the cumulative number of hours per week that you participated in amateur physical activity over the past year? ____hours/week

7.9 What is the average cumulative number of hours per week that you engage in similar strenuous physical activity? ____ hours/week

7.10 How many time do you spend on various household chores (including childcare) on average every day? ____ hours/day

7.11 How many hours per day do you spend in static activities (including watching TV, mobile phone /Pad, reading, eating, gathering, playing chess, CARDS, playing video games, surfing the Internet, knitting, etc.)？ ___ hours/day
