# Supplementary material for: Association between Dietary Inflammatory Index and Type 2 diabetes mellitus in Xinjiang Uyghur autonomous region, China
Source: PeerJ. 2021 Jul 16;9:e11159. doi: 10.7717/peerj.11159 (PMC8288110; doi:10.7717/peerj.11159)
Supplement: File S4 [file peerj-09-11159-s004.pdf]

国家重点研发计划“精准医学研究”重点专项

西北区域自然人群队列研究

新疆多民族自然人群队列建设及

健康随访研究

课题调查问卷

体检编号：□□□□□□□□□

国家重点研发计划“精准医学研究”重点专项西北区域自然人群队列研究

新疆多民族自然人群队列建设及健康随访研究

基线调查问卷

姓名：		性别：①男性 ②女性
年龄：	——岁	调查日期: __年__月__日

调查流程

编号	内容	完成后打√签字
1	登记领表、知情同意	□
2	采集血标本	□
3	测量血压	□
4	测量身高、腰围	□
5	测量体重、体脂	□
6	问诊调查	□
7	收表检查	□

体格检查项目

**1.1** 身高（**cm**） — — —.—<br>**1.2** 腰围（**cm**） — — —.—	**1.5** 体重（**kg**） — — —.—
**1.3 心率**（次/分） — — —<br>**1.4** 血压（**mmHg**）<br>收缩压 舒张压<br>第 1 次— — —.— — — —.—<br>第 2 次— — —.— — — —.—	**1.6** 体脂成分<br>体脂肪率（**%**） — — —.—<br>体脂肪量（**kg**） — — —.—<br>肌肉量（**kg**） — — —.—

一、一般信息（请在相应的选项○上打✓，请在□内按要求填写内容）

1.1 体检编号：（见封面）

1.2 你所在单位的名称：____________________

1.3 性别：（见封面） ○ 男 ○ 女

1.4 出生日期：□□□□年□□月□□日

1.5 身份证号：□□□□□□□□□□□□□□□□□□

1.5.1 医保编号：□□□□□□□□□□□□□□□□□□

1.6 家庭住址：新疆维吾尔 自治区 （市/自治州） ____________________街道

/乡镇____________________居委会**/**村

1.7 你的手机号码： □□□ □□□□ □□□□

1.7.1 重要联系人手机号码： □□□ □□□□ □□□□

1.8 你的民族

○ 汉族 ○ 回族

○ 维吾尔族 ○ 哈萨克族

○ 其他（**1.8a**） ____________

1.9 你所接受过的最高教育是什么？

○ 未正规上过学 ○ 大专

○ 小学 ○ 大学

○ 初中 ○ 研究生以上

○ 高中（包括中专/技校）

1.10 去年你全家一年的总收入（包括各种来源）约为多少？

○ <2,500 元 ○ 3.5 万-4.99 万元

○ 2,500-4,999 元 ○ 5 万-7.4 9 万元

○ 5,000-9,999 元 ○ 7.5 万-9.99 万元

○ 1 万-1.99 万元 ○ 10 万-19.9 万元

○ 2 万-3.49 万元 ○ ≥20 万元

二、饮茶及咖啡情况（请在相应的选项 ○上打√，请在□内按要求填写内容）

2.1 在过去一年里，你大概多长时间喝一次茶？

○ 从不或几乎从不喝

○ 只在特殊场合下（如节假日或做客时）偶尔喝

○ 一年当中只在特殊季节喝（如农忙或夏季）

○ 一年当中不分季节，每个月都喝，但频度不到每周一次

○ 一年当中不分季节，基本上每周都喝

2.2 在过去一年里，你大概多长时间喝一次咖啡？

○ 从不或几乎从不喝咖啡

○ 只在特殊场合下（如节假日或做客时）偶尔喝

○ 每月都喝，但频度不到每周一次

○ 基本上每周都喝

2.3 你大约从多大年龄开始养成每周都喝咖啡的习惯？ □□岁

三、饮酒情况（请在相应的选项 ○上打√）

3.1 在过去一年里，你大概多长时间喝一次酒？

○ 从不或几乎从不喝酒

○ 只在特殊场合下（如喜庆或节假日）偶尔喝

○ 一年当中只在特定几个月里饮酒（如农忙或夏季），而其他季节一般不喝酒

○ 一年当中不分季节，每个月都喝，但频度不到每周一次

○一年当中不分季节，基本上每周至少喝一次酒

四、吸烟情况（请在相应的选项 ○上打√，请在□内按要求填写内容）

4.1 你现在多长时间吸一次烟？

○ 不吸烟　○偶尔吸　○大部分天数吸　○每天都吸

4.2 若你过去曾经吸烟现在已经不吸，那你戒烟有多久了？ □□年□□月

4.3 促使你戒烟的一个最主要的原因是什么？（请选择一项最主要的回答）

○ 因为所患的疾病

○ 家人反对

○ 担心影响今后的健康（尚未患病）

○ 医生建议

○ 因经济负担过重

○ 其它_

4.4 你大约从几岁开始养成每天或基本上每天都吸烟的习惯？ □□岁

4.5 你是否曾经尝试过戒烟（需至少坚持 1 周才算）？

○ 是 ○否

五、膳食情况（请在相应的选项 ○上打√，请在____内按要求填写内容）

5.1 在过去一年里，你大概多久食用一次下列食物或饮品？

(请在每行选择一个○打√，并填写摄入量，如摄入量不清楚，请录入999)

**主食**	每天	4-6次/周	1-3次/周	1-3次/月	不吃或极少吃	你食用时，每次所摄入的量是多少?
大米	○	○	○	○	○	________两/次
小米	○	○	○	○	○	________两/次
面条	○	○	○	○	○	________两/次
馒头	○	○	○	○	○	________两/次
油饼	○	○	○	○	○	________两/次
油条	○	○	○	○	○	________两/次
杂粮（玉米、高粱、小米、青稞等）	○	○	○	○	○	________两/次

**动物性食物**	每天	4-6次/周	1-3次/周	1-3次/月	不吃或极少吃	每次摄入量
大肉	○	○	○	○	○	________两/次
羊肉	○	○	○	○	○	________两/次
羊肉串	○	○	○	○	○	________两/次
牛肉	○	○	○	○	○	________两/次
牛肉干	○	○	○	○	○	________两/次
马肉	○	○	○	○	○	________两/次
家禽及制品	○	○	○	○	○	________两/次
鸡肉	○	○	○	○	○	________两/次
鸭肉	○	○	○	○	○	________两/次
鸽子肉	○	○	○	○	○	________两/次
水产/海鲜品	○	○	○	○	○	________两/次
大虾	○	○	○	○	○	________两/次
鱼（标注种类）	○	○	○	○	○	________两/次
蛋类及制品	○	○	○	○	○	________两/次
鸡蛋	○	○	○	○	○	________两/次
鸭蛋	○	○	○	○	○	________两/次
鹌鹑蛋（五香）	○	○	○	○	○	________两/次
动物内脏类	○	○	○	○	○	________两/次
羊肝	○	○	○	○	○	________两/次
牛肝	○	○	○	○	○	________两/次
鸡肝	○	○	○	○	○	________两/次

**植物性食物**	每天	4-6次/周	1-3次/周	1-3次/月	不吃或极少吃	每次摄入量
新鲜蔬菜	○	○	○	○	○	________两/次
胡萝卜	○	○	○	○	○	________两/次
白萝卜	○	○	○	○	○	________两/次
红萝卜	○	○	○	○	○	________两/次
青萝卜	○	○	○	○	○	________两/次
豆角	○	○	○	○	○	________两/次
茄子	○	○	○	○	○	________两/次
西红柿	○	○	○	○	○	________两/次
辣椒	○	○	○	○	○	________两/次

南瓜	○	○	○	○	○	________两/次
大蒜	○	○	○	○	○	________两/次
大葱	○	○	○	○	○	________两/次
洋葱（皮牙子）	○	○	○	○	○	________两/次
姜	○	○	○	○	○	________两/次
姜黄	○	○	○	○	○	________两/次
大白菜	○	○	○	○	○	________两/次
西兰花	○	○	○	○	○	________两/次
菠菜	○	○	○	○	○	________两/次
香菜	○	○	○	○	○	________两/次
胡椒	○	○	○	○	○	________两/次
新鲜瓜果	○	○	○	○	○	________两/次
苹果	○	○	○	○	○	________两/次
梨	○	○	○	○	○	________两/次
葡萄	○	○	○	○	○	________两/次
橙子	○	○	○	○	○	________两/次
香蕉	○	○	○	○	○	________两/次
土豆红薯类	○	○	○	○	○	________两/次
土豆	○	○	○	○	○	________两/次
粉条	○	○	○	○	○	________两/次
红薯	○	○	○	○	○	________两/次
豆类及豆制品（黄豆，豆腐）	○	○	○	○	○	________两/次
黄豆	○	○	○	○	○	________两/次
豆奶粉	○	○	○	○	○	________两/次
豆腐	○	○	○	○	○	________两/次
豆腐皮	○	○	○	○	○	________两/次
绿豆	○	○	○	○	○	________两/次
蚕豆（大豆）	○	○	○	○	○	________两/次
鹰嘴豆	○	○	○	○	○	________两/次

**奶制品**	**每天**	**4-6次/周**	**1-3次/周**	**1-3次/月**	**不吃或极少吃**	**每次摄入量**
牛、羊、马奶	○	○	○	○	○	______毫升/次
牛奶	○	○	○	○	○	______毫升/次
马奶	○	○	○	○	○	______毫升/次
羊奶	○	○	○	○	○	______毫升/次
酸奶（凝固/液态）	○	○	○	○	○	______毫升/次
其它奶制品（奶粉、奶酪, 黄油等）	○	○	○	○	○	______毫升/次
奶疙瘩	○	○	○	○	○	________两/次
奶油	○	○	○	○	○	________两/次
酥油	○	○	○	○	○	________两/次
奶皮子	○	○	○	○	○	________两/次
酥油茶	○	○	○	○	○	______毫升/次
奶茶	○	○	○	○	○	______毫升/次

**其它食物**	**每天**	**4-6次/周**	**1-3次/周**	**1-3次/月**	**不吃或极少吃**	**每次摄入量**
咸菜泡菜	○	○	○	○	○	________两/次
干菜（包括蘑菇、木耳等）	○	○	○	○	○	________两/次

坚果	○	○	○	○	○	________两/次
核桃	○	○	○	○	○	________两/次
巴旦木	○	○	○	○	○	________两/次
花生	○	○	○	○	○	________两/次
红枣	○	○	○	○	○	________两/次
枸杞	○	○	○	○	○	________两/次
葡萄干	○	○	○	○	○	________两/次
糖果及巧两力	○	○	○	○	○	________两/次
糕点（蛋糕、饼干、冰激凌等）	○	○	○	○	○	________两/次

**软性饮品**	**每天**	**4-6次/周**	**1-3次/周**	**1-3次/月**	**不吃或极少吃**	**每次摄入量**
豆浆	○	○	○	○	○	________毫升/次
纯果汁/蔬菜汁（无加糖）	○	○	○	○	○	________毫升/次
碳酸饮料（可乐、汽水）	○	○	○	○	○	________毫升/次
其他含糖饮料	○	○	○	○	○	________毫升/次
酒（注明类别）	○	○	○	○	○	________毫升/次
咖啡	○	○	○	○	○	________毫升/次
红/绿茶	○	○	○	○	○	________毫升/次

**菌藻类**	**每天**	**1-3次/周**	**4-6次/周**	**1-3次/月**	**不吃或极少吃**	**每次摄入量**
金针菇	○	○	○	○	○	________两/次
牛菌菇	○	○	○	○	○	________两/次
蘑菇	○	○	○	○	○	________两/次
木耳	○	○	○	○	○	________两/次
香菇	○	○	○	○	○	________两/次
海带	○	○	○	○	○	________两/次
紫菜	○	○	○	○	○	________两/次

5.2 与你的朋友或同事相比，你所喜欢的口味如何？

○ 口味很清淡　　○ 不咸不淡　　○ 口味偏咸

5.3 在过去的一年中，下面列举的食物/饮食情况你大约多久出现一次？

特色食物	每天	4-6次/周	1-3次/周	1-3次/月	从不或极少	每次摄入量
馕	○	○	○	○	○	____两/次
八宝茶	○	○	○	○	○	__毫升/次
药茶	○	○	○	○	○	__毫升/次
藏红花	○	○	○	○	○	____两/次
**油脂**	**每天**	**4-6次/周**	**1-3次/周**	**1-3次/月**	**从不或极少**	
动物油	○	○	○	○	○	____两/次
菜籽油	○	○	○	○	○	____两/次
花生油	○	○	○	○	○	____两/次
豆油	○	○	○	○	○	____两/次
胡麻油	○	○	○	○	○	____两/次
调和油	○	○	○	○	○	____两/次
葵花籽油	○	○	○	○	○	____两/次
玉米油	○	○	○	○	○	____两/次
橄榄油	○	○	○	○	○	____两/次
茶籽油	○	○	○	○	○	____两/次
**特定饮食行为**	**每天**	**4-6次/周**	**1-3次/周**	**1-3次/月**	**从不或极少**	
吃零食	○	○	○	○	○	
吃方便面/方便米线等方便食品	○	○	○	○	○	
吃宵夜	○	○	○	○	○	
吃熏肉/腊肉	○	○	○	○	○	
吃火腿肠/香肠等加工肉食	○	○	○	○	○	
不吃早餐	○	○	○	○	○	
吃油炸食品	○	○	○	○	○	
吃烧烤类食物	○	○	○	○	○	
吃西式快餐(如比萨饼、汉堡等)	○	○	○	○	○	
在餐馆或小吃店吃饭	○	○	○	○	○	

5.4　在过去一年里，你是否服用过下列营养品并且持续服用时间至少达一个月？

否	是	
○	○	鱼肝油/鱼油
○	○	维生素
○	○	钙/铁/锌
○	○	人参类
○	○	其它保健补品

5.5　在过去一年里，你大概多久吃一次辣食？

○ 从不/几乎从不吃辣
○ 偶尔吃，但不到每周一次
} ➔若选此两项，请转至问题 **5.10**

○ 每周 1-2 次

○ 每周 3-5 次

○ 每天或几乎每天都吃

5.6 你大约从几岁开始养成每周吃辣食的习惯？ ____岁

5.7 在过去一年里，你吃饭时食醋的频率大概是多久一次？

○ 从不/几乎从不吃醋
○ 偶尔吃，但不到每周一次
} ➔若选此两项，请转至问题 **6.1**

○ 每周 1-2 次

○ 每周 3-5 次

○ 每天或几乎每天都吃

5.8 你的食醋习惯大约是从几岁开始养成的？ ____岁

5.9 你认为自己对醋的偏好程度怎样？

○ 特别喜欢，量大 ○ 比较喜欢，量中等 ○ 一般，量少 ○ 反感，不吃

六、个人及家庭健康状况（请在相应的选项 ○上打√，请在_____内按要求填写内容）

6.1 你目前的健康状况如何?

6.1.1 自我评价情况？
○良好
○ 较好
○ 一般
○ 较差

6.1.2 和同龄人相对比情况？
○更好
○相似
○较差
○不知道

6.2 在过去一年里，你一共因病住过几次院？（如无请填 0 0） ☐☐次

6.3 你是否曾被乡/区级或以上医院医生诊断患有过下列疾病?(请在每项☑回答, 如有, 请作进一步说明)
是 否 ☐ ☐

疾病名称	是否患病？	首次诊断时年龄？	是否接受治疗？	目前是否仍接受治疗？	是否住过院？	如是，上次住院日期
糖尿病	○	_______	○	○	○	_______
急性心梗（冠心病的一种）	○	_______	○	○	○	_______
心绞痛（冠心病的一种）	○	_______	○	○	○	_______
其它缺血性心脏病中风/小卒中发作	○	_______	○	○	○	_______

高血压	○	______	○	○	○	______
肺心病	○	______	○	○	○	______
风湿性心脏病	○	______	○	○	○	______
肺结核	○	______	○	○	○	______
肺气肿	○	______	○	○	○	______
慢性支气管炎	○	______	○	○	○	______
慢阻肺	○	______	○	○	○	______
哮喘	○	______	○	○	○	______
慢性肝炎/肝硬化	○	______	○	○	○	______
消化道溃疡	○	______	○	○	○	______
胆结石/胆囊炎	○	______	○	○	○	______
慢性肾病	○	______	○	○	○	______
骨质疏松症	○	______	○	○	○	______
骨折	○	______	○	○	○	______
风湿性关节炎	○	______	○	○	○	______
抑郁	○	______	○	○	○	______
焦虑	○	______	○	○	○	______
神经衰弱	○	______	○	○	○	______
其它精神心理疾患	○	______	○	○	○	______
脑外伤	○	______	○	○	○	______
其他 1	○	______	○	○	○	______
其他 2	○	______	○	○	○	______
恶性肿瘤（癌症）*	○	______	○	○	○	______

*如有肿瘤，请在方框内注明具体部位：（如多于一处，请选首发的肿瘤部位）

□肺，□食道，□胃，□肝，□肠，□乳腺，□前列腺，□宫颈，□其它

七、体力活动情况（请在相应的选项 ○上打√，请在_____内按要求填写内容）

7.1 在过去一年里，你上班时是以静坐、站立或干体力活为主？

○ 以静坐为主（如行政管理人员，秘书等）

○ 以站立为主（如营业员，门卫等）

○ 以一般体力活为主（常温下不太出汗如管道工、电工、木工、泥工等）

○ 以重体力劳动为主（常温下很容易出汗如装卸、采矿、炼钢等）

○ 离退休或家务、待业一年以上或肢体残疾无法正常劳动

7.2　你每周平均工作多少天？　　______天

7.3　你每天平均工作多少小时？　______小时

7.4　在过去一年里，你通常采用什么方式上下班或外出干活？

○ 步行　　○ 骑摩托车

○ 骑自行车　　○ 私家车/出租

○ 公共交通（公交车，地铁，渡船）

○ 　通常在家里或在家附近上班

7.5　你外出工作/干活时，每天花在路上的往返时间有多长？　　______分钟

7.6　在过去一年里，你业余时间一般多久参加一次体育锻炼？

○ 从不或几乎从不参加

○ 每月 1-3 次

○ 每周 1-2 次

○ 每周 3-5 次

○ 每天或几乎每天都锻炼

7.7　你最常用的锻炼方式是哪一种？

○ 太极拳/气功/散步/瑜伽

○ 跑步/健美操

○ 球类运动（篮球、乒乓球、羽毛球等）

○ 快走/扭秧歌/广场舞

○ 游泳

○ 其它（如爬山、骑行、跳绳、踢毽子等）

7.8 在过去一年里，你每周参加业余体育锻炼的累计时间有多长？　　______小时/周

7.9 你平均每周从事类似强体力活动的累计时间大约有多长？　　______小时/周

7.10 你平均每天做各类家务活（包括带孩子）的时间有多长？　　______小时/天

7.11 你平均每天业余时间用于静态活动（包括看电视、手机/Pad，读书报，吃饭，聚餐，下棋，打牌，玩电子游戏，上网及编织等）的时间一共有多长？　　______小时/天
