# Supplementary material for: Association between Dietary Inflammatory Index and Type 2 diabetes mellitus in Xinjiang Uyghur autonomous region, China
Source: PeerJ. 2021 Jul 16;9:e11159. doi: 10.7717/peerj.11159 (PMC8288110; doi:10.7717/peerj.11159)
Supplement: File S5 [file peerj-09-11159-s005.pdf]

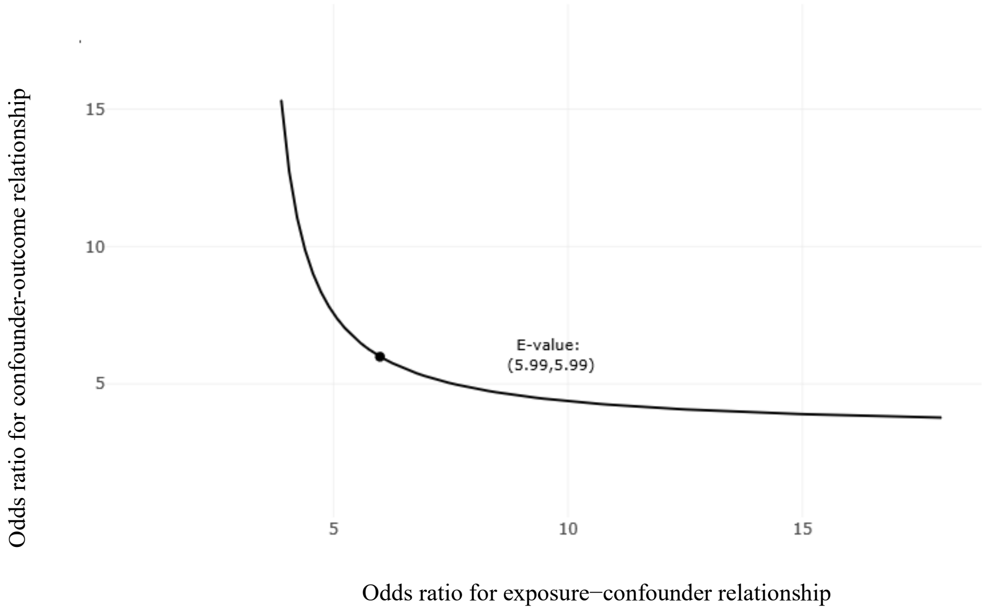

Figure 4: To compute the E-value for this odds ratio.

We input the point estimate (3.27) and the lower and upper confidence limits (2.38 and 4.50, respectively).
