# Supplementary material for: Association between Dietary Inflammatory Index and Type 2 diabetes mellitus in Xinjiang Uyghur autonomous region, China
Source: PeerJ. 2021 Jul 16;9:e11159. doi: 10.7717/peerj.11159 (PMC8288110; doi:10.7717/peerj.11159)
Supplement: File S6 [file peerj-09-11159-s006.docx]

| Variables | DII quintiles | | | *P*-interaction |
| --- | --- | --- | --- | --- |
|  | Q1 | Q5 | |  |
|  | OR (95%CI) | OR (95%CI) | *P*-Value |  |
| Activity |  |  |  | <0.001 |
| Low | 1 | 5.62(3.00,10.51) | <0.001 |  |
| Medium and High | 1 | 2.39(1.64,3.49) | <0.001 |  |
| BMI(kg/m^2^) |  |  |  | <0.001 |
| <24 | 1 | 2.42(1.34,4.40) | <0.05 |  |
| 24-28 | 1 | 2.78(1.68,4.58) | <0.001 |  |
| >28 | 1 | 4.62(2.50,8.56) | <0.001 |  |
| Ethnicity |  |  |  | <0.001 |
| Uyghurs | 1 | 4.11(1.85,9.14) | 0.001 |  |
| Hui | 1 | 3.80(2.13,6.77) | <0.001 |  |
| Han | 1 | 3.69(1.80,7.59) | <0.001 |  |
| Kazak | 1 | 2.23(0.87,7.59) | 0.10 |  |
| Others | 1 | 1.29(0.61,2.74) | 0.51 |  |
| Age(years) |  |  |  | <0.001 |
| ≥55 | 1 | 5.14(2.79,9.47) | <0.001 |  |
| <55 | 1 | 2.27(1.41,3.67) | <0.05 |  |

**Table 4：**

**Stratified analysis of the association between DII and T2DM after adjusting for potential confounding factors (Quartile 5 vs. Quartile 1)**
